# IL-10-dependent partial refractoriness to Toll-like receptor stimulation modulates gut mucosal dendritic cell function

**DOI:** 10.1002/eji.200737909

**Published:** 2008-06

**Authors:** Ivan Monteleone, Andrew M Platt, Elin Jaensson, William W Agace, Allan McI Mowat

**Affiliations:** 1 Division of Immunology Infection and Inflammation, Glasgow Biomedical Research Centre, University of GlasgowGlasgow, Scotland; 2 Immunology Section, Lund UniversityBMC I13, Lund, Sweden

**Keywords:** Dendritic cells, Mucosa, Tolerance, IL-10, TLR

## Abstract

The default response of the intestinal immune system to most antigens is the induction of immunological tolerance, which is difficult to reconcile with the constant exposure to ligands for TLR and other pattern recognition receptors. We showed previously that dendritic cells (DC) from the lamina propria of normal mouse intestine may be inherently tolerogenic and here we have explored how this might relate to the expression and function of Toll-like receptors (TLR). Lamina propria (LP) DC showed higher levels of TLR 2, 3, 4 and 9 protein expression than spleen and MLN DC, with most TLR-expressing DC in the gut being CD11c^lo^, class II MHC^lo^, CD103^–^, CD11b^–^ and F4/80^–^. TLR expression by lamina propria DC was low in the upper small intestine and higher in distal small intestine and colon. Freshly isolated lamina propria DC expressed some CD40, CD80, CD86 and functional CCR7. These were up-regulated on CD11c^lo^, but not on CD11c^hi^ LP DC by stimulation *via* TLR. However, there was little induction of IL-12 by either subset in response to TLR ligation. This was associated with constitutive IL-10 production and was reversed by blocking IL-10 function. Thus, IL-10 may maintain LP DC in a partially unresponsive state to TLR ligation, allowing them to have a critical role in immune homeostasis in the gut.

## Introduction

The intestine has a vast surface area, which is exposed continuously to dietary antigens and commensal micro-organisms, as well as an array of different pathogens. Although the intestinal immune system generates strong protective immunity against invading organisms, responses to harmless antigens are tightly regulated, so that unnecessary inflammation is prevented and tolerance is induced 1. In most compartments of the immune system, this decision is determined by the way in which dendritic cells (DC) present antigen to T lymphocytes. After infection by pathogenic organisms or during inflammation, DC are activated to express a full range of costimulatory molecules and pro-inflammatory cytokines, ensuring the efficient activation of effector T cells. However, DC also play a critical role in the induction of tolerance to both self and foreign antigens, often because they are in a “quiescent” state which allows them to present peptide-MHC to specific T cells in the absence of full costimulation 2.

Recent evidence has shown that there are many DC with this “quiescent” phenotype in the intestine and its lymphoid organs. Subsets of DC from Peyer's patches (PP), mesenteric lymph nodes (MLN) and mucosal lamina propria (LP) have also been found to produce regulatory cytokines such as IL-10 and TGFβ and to be capable of polarising T cells towards a regulatory or anti-inflammatory phenotype 1, 3. Although the exact role of each of these populations in tolerance to individual forms of intestinal antigen remains to be clarified, our own and other previous studies indicated that DC in the LP are particularly important after feeding soluble proteins 3, 4. A substantial proportion of such antigens is taken up by these DC *in situ* and can then be presented to naive T cells, probably after CCR7-dependent migration to the draining MLN and transfer of *in vivo* loaded LP DC induces specific tolerance in naive recipients 3, 5, 6. Together, these findings highlight the possibility that under physiological conditions, immunomodulatory DC predominate in the LP and are important for maintaining intestinal homeostasis by preventing the induction of inflammatory T cells.

However, these results are somewhat paradoxical in view of the fact that mucosal DC are in an environment rich in bacterial products and especially with the recent evidence that LP DC can contact and engulf luminal bacterial directly by extending processes across the epithelial layer 7–9. Commensal bacteria can also be found in DC in gut-associated lymphoid organs 10 and it is not at all clear why this constant interplay between the resident flora and local DC does not provoke inflammation. DC use a variety of molecules to sense microbial agents, of which the best understood are the family of Toll-like receptors (TLR) 11. Recognition of conserved pathogen-associated molecular patterns (PAMP) by TLR normally induces activation of DC, and in our previous work, we showed that the TLR 4 ligand bacterial lipopolysaccharide (LPS) induced up-regulation of CD40, CD80 and CD86 by LP DC. However, DC isolated from LP or intestinal lymph fail to produce nitric oxide or mRNA for IL-12p40 in response to LPS, suggesting that mucosal DC may be partially refractory to activation *via* TLR 3, 12, 13. Indeed, recent studies suggest that TLR signalling actively down-regulates inflammation in the intestine and are crucial to maintain intestinal immune homeostasis 14–19.

Here, we have examined if aberrant responsiveness of LP DC to TLR ligands could account for their ability to recognise local microbes without inducing inflammation by investigating the expression and functions of a variety of TLR on DC isolated from the intestine of normal mice. Our results show that although these DC express high levels of several TLR and can up-regulate costimulatory molecules and CCR7 in response to appropriate TLR ligands, their secretion of pro-inflammatory cytokines is inhibited by constitutive production of IL-10. Selective regulation of TLR responsiveness may play a critical role in allowing local microorganisms to be screened by mucosal DC without the risk of unnecessary inflammation.

## Results

### Expression of TLR by DC in lamina propria, spleen and MLN

**Figure 1:**
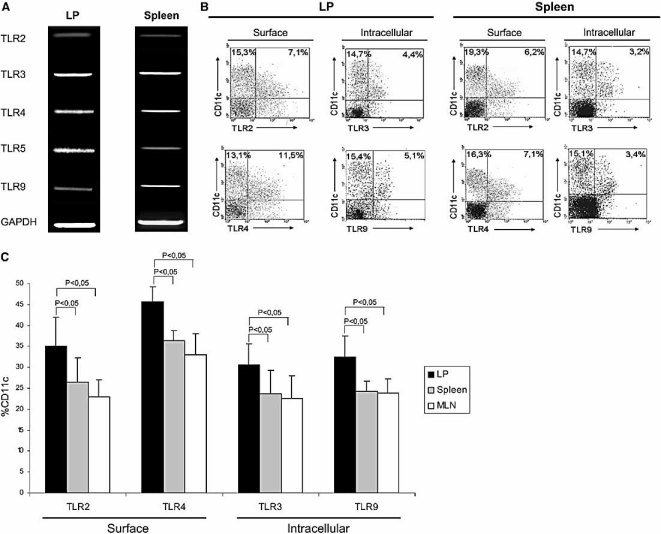
TLR expression by DC in LP, spleen and MLN. (A) mRNA for TLR2, 3, 4, 5, 9 and GAPDH was identified by RT-PCR using MACS-purified CD11c^+^ cells isolated by enzymatic digestion from the small intestinal LP and spleen. The results are representative of two repeat experiments. (B) Representative flow cytometric analysis showing the surface expression of TLR2 and 4 and the intracellular expression of TLR3 and 9 by small intestinal LP and spleen DC. Cells were isolated by enzymatic digestion, stained with FITC or PE antibodies anti-CD11c and FITC or PE antibodies anti-TLR, with dead cells gated out by staining with PI. The data shown are the proportion of live gated, CD11c^+^ cells positive for each marker. (C) Proportions of DC expressing surface TLR2 or 4, and intracellular TLR3 or 9 in LP (black), spleen (grey) and MLN (white) as assessed by flow cytometric analysis as described in (B). The data shown are the percentages of TLR^+^ cells out of live-gated (PI^–^) CD11c^+^ cells in each tissue and are the mean + 1 SD of three separate experiments.

In the first experiments, we analysed the expression of the various TLR by DC from different tissues. Non-quantitative PCR analysis showed expression of mRNA for TLR 2, 3, 4, 5 and 9 in MACS-purified CD11c^+^ cells from LP (Fig. 1A). A similar pattern was found in spleen DC, indicating that both peripheral and mucosal DC are capable of synthesising all TLR.

We next determined the expression of TLR proteins by DC from the different sites. While some TLR (TLR 1, 2, 4, 5 and 6) are expressed on the cell surface, others (TLR 3, 7, 8, and 9) are found almost exclusively in intracellular compartments such as endosomes, where they interact with internalised ligands such as nucleic acids 11. Therefore, we analysed the expression of TLR 3 and 9 in DC by intracellular staining and used surface staining to assess TLR 2 and 4 expression (Fig. 1B and C). Significant numbers of both spleen and LP DC expressed TLR 2, 3, 4 and 9 proteins, as assessed by flow cytometry (Fig. 1B). Overall, significantly more LP DC expressed TLR than in spleen, with the highest expression being found for TLR 4, which was present on more than 40% of LP DC (Fig. 1C). TLR expression by MLN DC was generally more similar to spleen than LP (Fig. 1C). No antibodies are currently available to assess TLR 7 or 8 expression by flow cytometry and we were unable to detect expression of TLR 5 using a selection of different commercial antibodies (data not shown).

### TLR expression by phenotypic and anatomical subsets of mucosal DC

**Figure 2:**
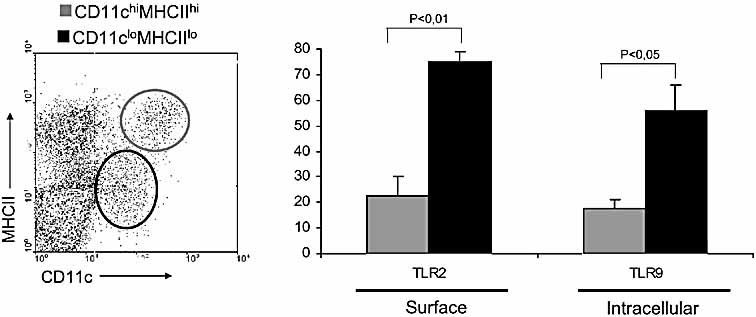
Two major sub-populations of CD11c^lo^ class II MHC^lo^ and CD11c^hi^ class II MHC^hi^ DC were identified among freshly isolated, enzymatically digested small intestinal LP cells (left panel). The proportions of live gated CD11c^lo^ class II MHC^lo^ (black) and CD11c^hi^ class II MHC^hi^ (grey) DC expressing surface TLR2 or intracellular TLR 9 are shown (right panel). The data shown are the percentages of TLR^+^ cells out of live-gated (PI^–^) CD11c^+^ cells in each tissue and are the mean ± 1 SD of three separate experiments.

**Figure 3:**
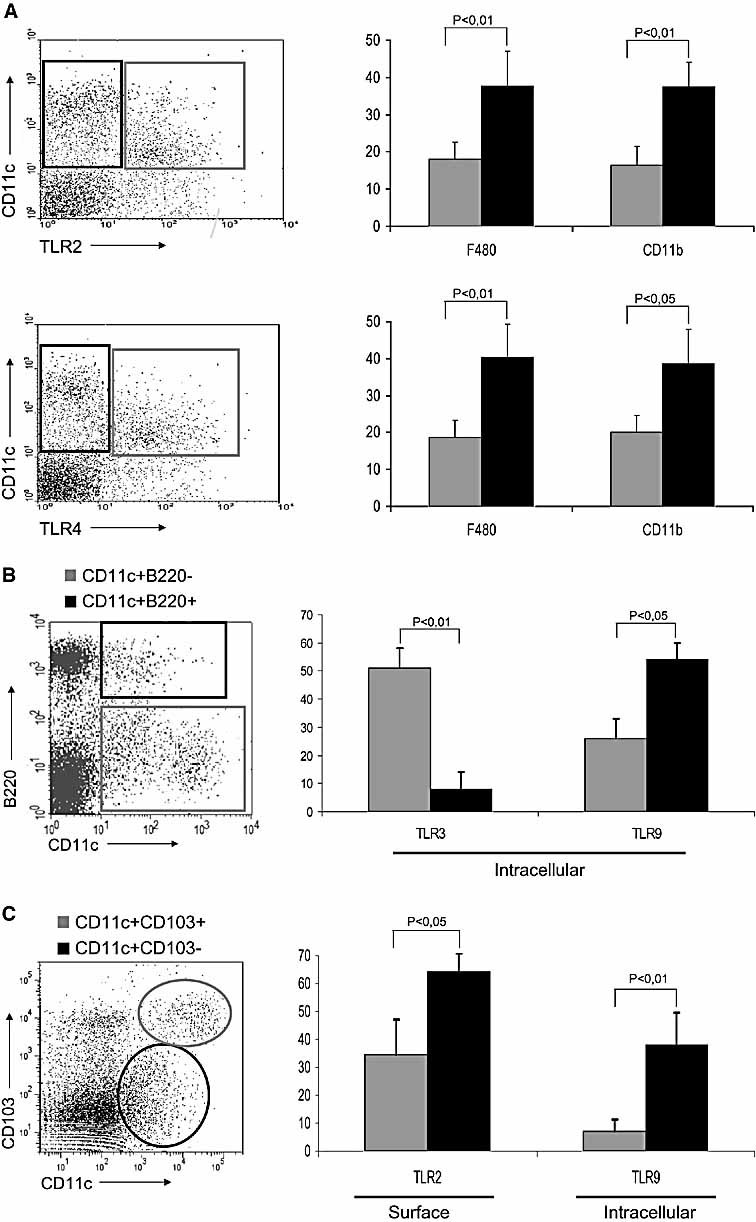
Distribution of TLR expression in different LP DC subsets. (A) TLR are expressed mainly by LP DC (CD11c^+^CD11b^–^F4/80^–^). CD11c^+^TLR2^–^ and CD11c^+^TLR2^+^ subsets of freshly-isolated LP cells were shown in the top left panel. The proportions of live-gated CD11c^+^TLR2^–^ (black) and CD11c^+^TLR2^+^ (grey) DC expressing surface F4/80 or CD11b are shown in the top right panel. CD11c^+^TLR4^–^ and CD11c^+^TLR4^+^ subsets of freshly-isolated LP cells were shown in the bottom left panel. The proportions of live-gated CD11c^+^TLR4^–^ (black) and CD11c^+^TLR4^+^ (grey) DC expressing surface F4/80 or CD11b are shown in the bottom right panel. (B) Expression of TLR by the CD11c^+^B220^–^ and CD11c^+^B220^+^ DC subsets within the small intestinal LP cells (left panel). The proportions of live-gated CD11c^+^B220^+^ (black) and CD11c^+^B220^–^ (grey) DC expressing intracellular TLR3 or TLR9 are shown (right panel). (C) Two subpopulation of DC CD11c^+^CD103^+^ (grey) and CD11c^+^ CD103^–^ (black) were found in freshly isolated small intestinal LP cells (left panel). The percentages of gated CD11c^+^CD103^+^ (grey) and CD11c^+^CD103^–^ (black) DC expressing intracellular TLR9 or surface TLR3 are shown (right panel). The data shown are the percentages of F4/80^+^, CD11b^+^ or TLR^+^ cells out of live-gated (PI^–^) CD11c^+^ cells in each tissue and are the mean + 1 SD of three separate experiments.

As we have shown previously 3, LP DC are a heterogeneous population and therefore we examined TLR expression by individual phenotypic subsets within these cells. First, we compared the expression of TLR on putative mature and immature LP DC. As described previously 3, a significant number of LP DC have the CD11c^lo^ class II MHC^lo^ phenotype typical of immature DC and many more of these cells expressed TLR 2 and 9 than the DC with the CD11c^hi^ class II MHC^hi^ phenotype of mature DC (75% and 55% for TLR 2 and 9 *vs.* 22.5% and 17.5%, respectively; Fig. 2). Recently, it has been suggested that some of the CD11c^+^ cells found in the small intestinal mucosa may be macrophage-like cells, as defined by expression of high levels of F4/80 20. In our hands, around 30% of total CD11c^+^ cells in LP expressed F4/80, but in contrast to the earlier study, most of these cells were also CD11c^hi^, suggesting these were mainly of the DC lineage (data not shown). Consistent with this, very few of the F4/80^+^ CD11c^+^ cells expressed TLR 2 and 4 compared with the CD11c^+^F4/80^–^ cells (18% and 17% *vs.* 49% and 60% for TLR 2 and 4, respectively; Fig. 3A). Interestingly, TLR expression was also lower on the CD11b^+^ subset of LP DC which contains most of the F4/80^+^ cells, than on the CD11c^+^CD11b^–^ subset (16% and 20% *vs.* 57% and 62% for TLR 2 and 4, respectively; Fig. 3A).

TLR 9 is characteristically expressed by plasmacytoid DC (pDC) 11 and we found previously that a substantial minority of DC in LP expressed B220, which is also present on pDC 3. Consistent with these being pDC, 95% of CD11c^+^B220^+^ DC from LP expressed the PDCA-1 marker (data not shown) and a significantly larger proportion of B220^+^ LP DC expressed TLR 9 than B220^–^ DC (55% *vs.* 25%; Fig. 3B). A further subpopulation of LP DC which has received recent attention is that expressing the epithelial associated integrin CD103 and which is responsible for converting naive T cells into gut homing Foxp3^+^ Treg cells 21–23. In our hands, the majority of CD11c^hi^ cells in LP were also CD103^+^, while CD11c^lo^ LP DC were CD103^–^ or CD103^lo^. Consistent with this, more of the CD103^–^ DC expressed TLR 2 than the CD103^+^ subset (64% *vs.* 34%; Fig. 3C) and very few CD103^+^ DC expressed TLR 9 (7% *vs.* 39%; Fig. 3C).

**Figure 4:**
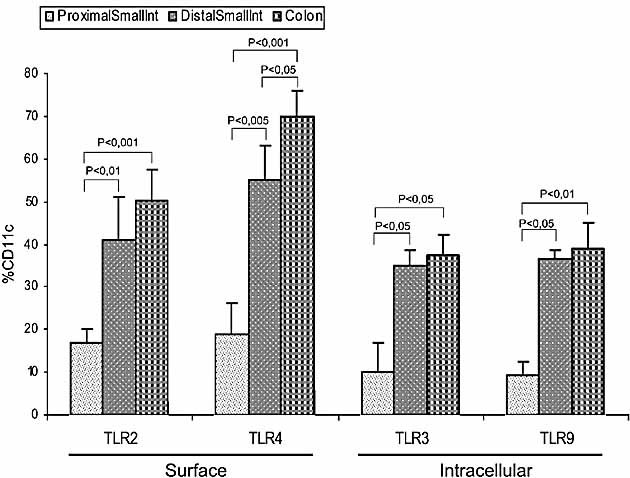
TLR expression on LP DC at different anatomical locations in the intestine. The proportions of freshly isolated, enzymatically digested small intestinal CD11c^+^ LP cells expressing surface TLR2 or 4, and intracellular TLR 3 or 9 in the proximal or distal small intestine or colon. All data are the mean + 1 SD of three separate experiments.

The fact that more mucosal DC expressed TLR than those in organised lymphoid tissues suggested that LP DC might be involved in interacting with local microorganisms. To examine this idea, we compared the expression of TLR on CD11c^+^ cells isolated from the LP of the upper jejunum, ileum and colon. As shown in Fig. 4, the expression of TLR 2, 3, 4 and 9 increased dramatically going down the intestine, with very low numbers found in the relatively sterile upper small bowel, compared with the distal small intestine and colon.

### TLR ligands induce phenotypic maturation of LP DC

In previous studies, we found that purified LP DC could up-regulate expression of class II MHC and costimulatory molecules in response to the TLR 4 ligand LPS, but failed to synthesise IL-12p40 mRNA after this stimulation 3. However, more recent work has suggested that LP DC from mouse small intestine may produce proinflammatory cytokines when stimulated with the TLR 5 ligand flagellin 24. To explore these discrepancies, we carried out a more detailed study of the responses of LP DC to a variety of TLR ligands.

**Figure 5:**
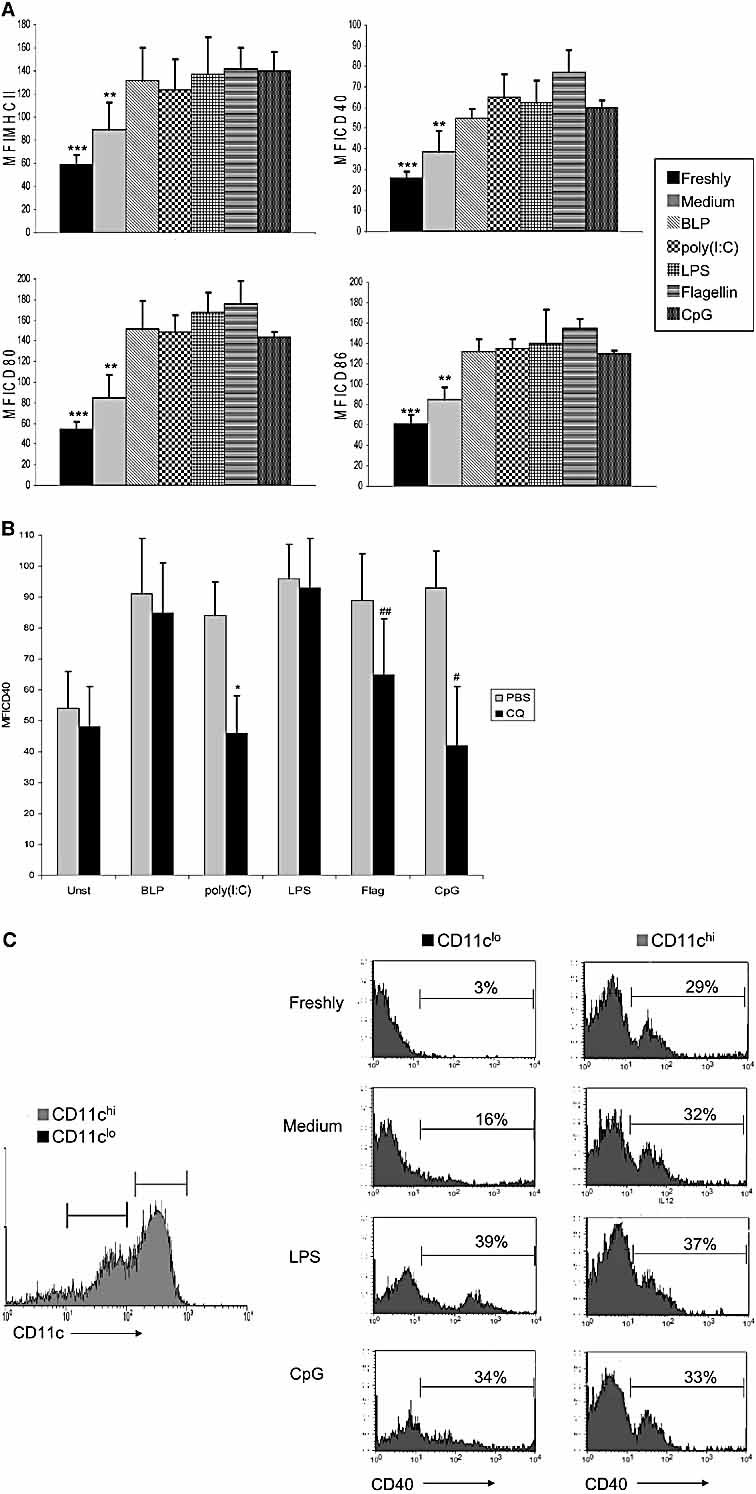
Effects of TLR ligation on the expression of activation markers by LP DC. (A) MACS-purified CD11c^+^ cells isolated by enzymatic digestion from the small intestinal LP were cultured with medium alone, or with different TLR ligands. After 12 h, DC were analysed for the expression of CD40, CD80, CD86 and class II MHC by flow cytometry. Freshly isolated DC were analysed in parallel as controls. The graph shows the MFI for each marker on live gated CD11c^+^ cells (***p≤0.05 *vs.* medium-treated and *p*<0.01 *vs.* all TLR ligands; **p≤0.05 *vs.* all TLR ligands). (B) Activation of DC by intracellular TLR requires endosomal acidification. MACS-purified CD11c^+^ LP DC were pre-treated with 2.5 μg/mL chloroquine (CQ, black) or PBS (grey) for 15 min and then stimulated for 12 h with different TLR ligands, prior to CD40 expression analysis by flow cytometry. The graph shows the MFI of CD40 expression on live gated CD11c^+^ cells (*p<0.005 *vs.* CD11c^+^ cells pretreated with PBS and stimulated with poly(I:C); ^#^*p*<0.01 *vs.* CD11c^+^ cells pre-treated with PBS and stimulated with CpG; ^##^*p*<0.05 *vs.* CD11c^+^ cells pretreated with PBS and stimulated with flagellin). The data are the mean + 1 SD of three separate experiments. (C) CD11c^lo^ and CD11c^hi^ LP DC differ in their response to TLR ligands. LP cells were FACS-sorted into CD11c^hi^ (grey line) and CD11c^lo^ (black line) subsets and the histograms show CD40 expression on freshly isolated cells, or after incubation in medium alone, LPS or CpG for 12 h.

Freshly isolated LP DC expressed variable but generally low levels of the maturation markers CD40, CD80 and CD86. Both the number of cells expressing these markers (Table 1) and the levels of their expression (Fig. 5A) increased somewhat after overnight culture in medium alone and even more markedly after addition of bacterial lipoprotein (BLP), poly I:poly C (poly (I:C)), LPS, flagellin or CpG, ligands for TLR 2, 3, 4, 5 and 9, respectively (Table 1 and Fig. 5A). Consistent with their intracellular localisation in acidic endosomes and as reported previously 25, the effects of ligating TLR 3 or TLR 9 on CD40 up-regulation were abolished by chloroquine (Fig. 5B). In contrast, the effects of ligating the surface expressed TLR 2 or 4 were not affected by chloroquine. The response to flagellin was partially inhibited, consistent with recent reports that flagellin may be recognised by both intracellular and extracellular receptors 26, 27. As TLR expression was markedly different on the CD11c^lo^ and CD11c^hi^ subsets of LP DC, we sorted these populations by FACS and compared their responses to TLR ligands. Around 30% of freshly isolated CD11c^hi^ DC expressed CD40 and this did not change after overnight culture in medium, LPS or CpG (Fig. 5C). In contrast, 10% or less of the CD11c^lo^ DC expressed CD40 when analysed immediately after isolation and these numbers increased markedly after overnight culture in medium, LPS or CpG, to levels equivalent to those found among CD11c^hi^ cells (Fig. 5C). Thus, the responsiveness of LP DC subsets to TLR stimulation correlates with their expression of the relevant receptors.

**Table 1:** Effects of TLR ligation on the expression of activation markers by LP DCa)

Stimulus	Class II MHC	CD40	CD80	CD86
Freshly isolated	66 ± 7%	25 ± 6%	50 ± 7%	42 ± 6%
Medium	76 ± 5%	50 ± 5%	64 ± 2%	70 ± 2%
BLP	88 ± 7%	71 ± 4%	85 ± 3%	85 ± 3%
LPS	86 ± 7%	67 ± 4%	83 ± 5%	80 ± 4%
Poly (I:C)	88 ± 3%	65 ± 4%	79 ± 3%	81 ± 7%
Flagellin	89 ± 3%	66 ± 4%	83 ± 6%	82 ± 4%
CpG	86 ± 8%	64 ± 3%	80 ± 5%	82 ± 6%

a) MACS-purified CD11c^+^ cells isolated by enzymatic digestion from the small intestinal LP were cultured with medium alone, or with BLP, poly(I:C), LPS, flagellin or CpG. After 12 h, the DC were analysed for the expression of CD40, CD80, CD86 and class II MHC by flow cytometry. Freshly isolated DC were analysed in parallel as controls. The data shown are the percentage of live-gated CD11c^+^ cells expressing each marker and are the means ± 1 SD of three separate experiments.

**Figure 6:**
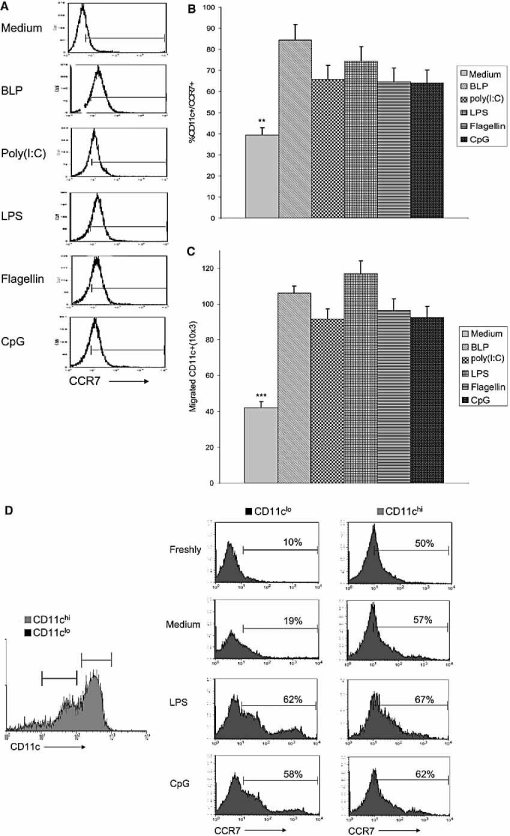
TLR stimulation enhances functional CCR7 expression by LP DC. (A) Representative flow cytometric analysis of CCR7 expression on live gated, MACS-purified CD11c^+^ cells from small intestinal LP stained with PE anti-CCR7 after stimulation for 6 h with medium alone, or with different TLR ligands. (B) Overall levels of expression of CCR7 on live gated, MACS-purified CD11c^+^ cells from small intestinal LP after stimulation for 6 h with medium alone or with different TLR ligands (**p<0.001 *vs.* BLP and LPS stimulation, *p*<0.005 *vs.* all other TLR stimulations). The data are the means + 1 SD of three separate experiments. (C) Chemotaxis of LP DC to CCR7 ligand CCL19. MACS-purified CD11c^+^ cells from small intestinal LP were cultured in medium, or with different TLR ligands for 4 h before being placed on the top of a transwell membrane and allowed to migrate in response to CCL19 (MIP-3β) in the bottom chamber. The number of CD11c^+^ cells which had migrated into the bottom chamber was determined by flow cytometry (***p≤0.001 *vs.* all TLR ligands). (D) CD11c^lo^ and CD11c^hi^ LP DC differ in their response to TLR ligands. LP cells were FACS-sorted into CD11c^hi^ (grey line) and CD11c^lo^ (black line) subsets and the histograms show CCR7 expression on freshly isolated cells, or after incubation in medium alone, LPS or CpG for 12 h.

An important aspect of maturation of tissue DC is up-regulation of CCR7 and for mucosal DC to function *in vivo*, they have to migrate to the draining MLN in a CCR7-dependent manner 5. Around 40% of LP DC expressed CCR7 when cultured in medium for 8 h and this was increased substantially by all the TLR ligands examined, with 70–80% becoming CCR7^+^ (Fig. 6A and B). This CCR7 was functional, as TLR-stimulated LP DC migrated much more efficiently in a chemotaxis assay *in vitro* in the presence of CCL19 (Fig. 6C). As with CD40, the expression of CCR7 and its induction by TLR ligation varied between the CD11c^hi^ and CD11c^lo^ subsets of LP DC. Freshly isolated CD11c^hi^ DC had higher levels of CCR7 than the equivalent CD11c^lo^ subset, but stimulation of CD11c^lo^ cells with LPS or CpG increased the expression of CCR7 to levels equivalent to those seen in the CD11c^hi^ subset, which showed no response to any of the stimuli (Fig. 6D). Thus, stimulation of LP DC with TLR ligands induces phenotypic maturation and allows the cells to become responsive to CCR7 ligands.

### LP DC have an IL-10-dependent inability to produce IL-12 in response to TLR ligands

**Figure 7:**
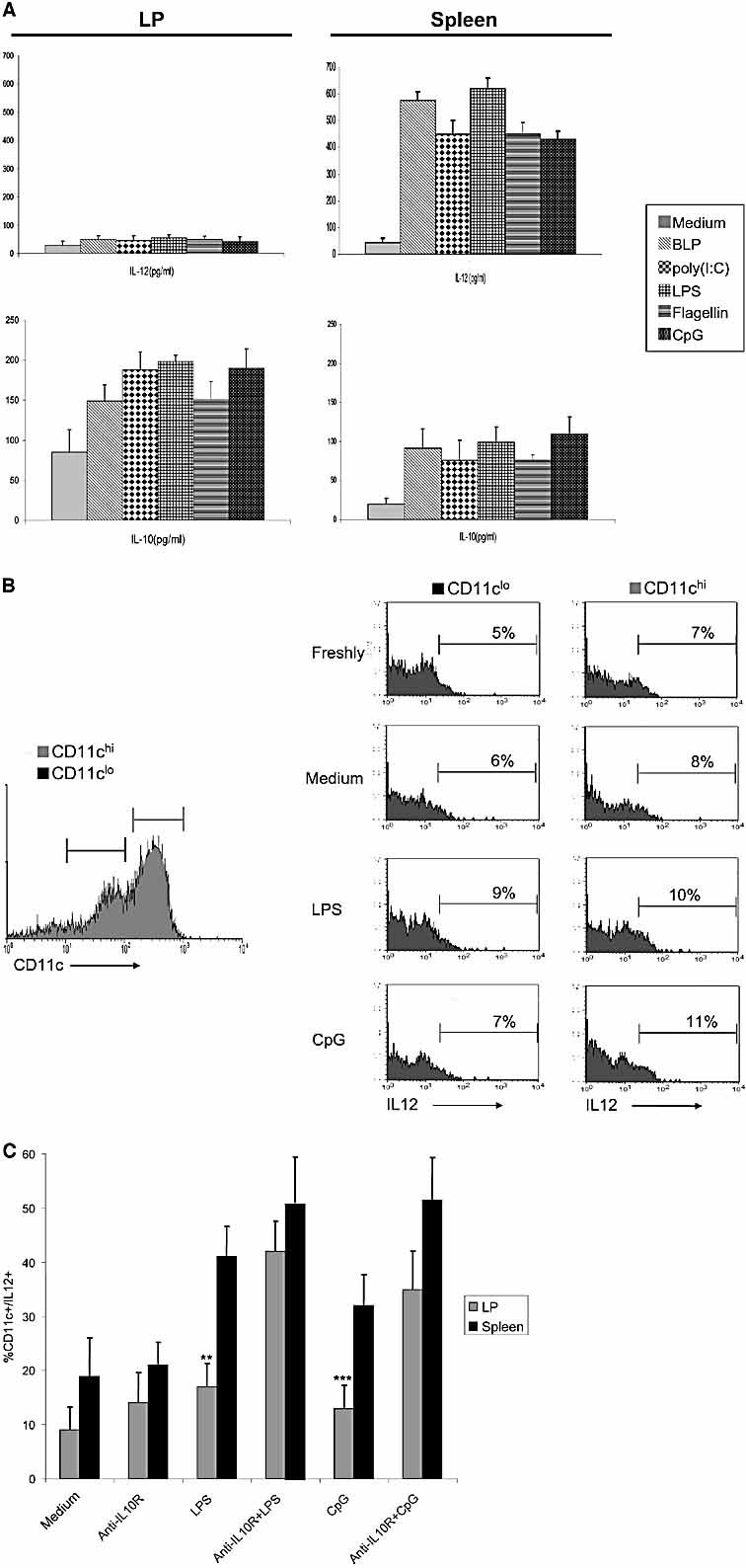
Constitutive production of IL-10 inhibits inflammatory cytokine production by LP DC in response to TLR stimulation. (A) Production of IL-12p70 (top) and IL-10 (bottom) by purified MACS-purified CD11c^+^ DC from small intestinal LP (left) or spleen (right) after culture with medium alone or with different TLR ligands for 24 h. Supernatants were analysed by sandwich ELISA and the results shown are pg/mL for duplicate cultures and are the means + 1 SD of three separate experiments. (B) Both CD11c^lo^ and CD11c^hi^ LP DC fail to produce IL-12 in response to TLR ligands. LP cells were FACS-sorted into CD11c^hi^ (grey line) and CD11c^lo^ (black line) subsets and the histograms show intracellular expression of IL-12p40 by freshly isolated cells, or after overnight incubation with medium alone, LPS or CpG for 6 h. (C) MACS-purified CD11c^+^ DC from small intestinal LP or spleen were pre-treated with medium alone or with 2 ng/mL of anti-IL10 receptor antibody for 60 min before being stimulated with TLR ligands for 6 h. Intracellular IL-12p70 levels were analysed by flow cytometry and the data shown are the percentage live gated CD11c^+^ cells positive for IL-12 and are the mean + 1 SD of three separate experiments. **p≤0.01 *vs.* LP DC preincubated with anti-IL10R and LPS; ***p<0.05 *vs.* LP DC preincubated with anti-IL10R and CpG.

In our previous work, we found that LP DC did not up-regulate IL-12p40 mRNA in response to LPS, but expressed IL-10 mRNA constitutively 3. We extended these findings here by measuring cytokine protein levels and by examining the responses to the range of TLR ligands we had found to produce phenotypic activation. Stimulation of purified LP DC with BLP, poly (I:C), LPS, flagellin or CpG did not induce secretion of IL-12p70 protein above the very low background levels found using unstimulated cells (Fig. 7A). Unlike the expression of phenotypic markers of activation, the CD11c^hi^ or CD11c^lo^ subsets of LP DC showed no differences in IL-12 production. In both cases, 10% or less of the cells stained positively for intracellular IL-12p40 under resting conditions and neither purified population showed any change after overnight culture in medium, LPS or CpG (Fig. 7B). In contrast, splenic DC produced high levels of IL-12 in response to all the TLR ligands. Unlike resting spleen DC, resting LP DC produced detectable amounts of IL-10 under resting conditions and these were further increased by TLR stimulation. TLR stimulated spleen DC also produced enhanced levels of IL-10 (Fig. 7A).

As IL-10 is a well-known inhibitor of IL-12 production 28, 29, we examined whether the inability of LP DC to secrete IL-12 was due to their constitutive production of IL-10. LP or spleen DC were therefore treated with anti-IL-10R before stimulation with either LPS or CpG and the numbers of IL-12-expressing cells determined by intracellular staining. As shown in Fig. 7C, spleen DC showed significant increases in IL-12-expressing cells after stimulation with LPS or CpG and these responses were increased further in the presence of anti-IL-10R. In contrast, LP DC again showed no significant response to LPS, CpG alone or with the rat IgG antibody control (data not show), but these responses were dramatically increased by treatment with anti-IL-10R (Fig. 7C).

Thus, constitutive production of IL-10 may be responsible for maintaining LP DC in a partially unresponsive state to TLR ligation.

## Discussion

The results presented here show that DC from normal small intestinal mucosa express a wide variety of intra-cellular and surface TLR, whose pattern of expression is determined by anatomical location and phenotypic subset. These TLR are functional, but are partially refractory to ligation, up-regulating costimulatory molecules and CCR7, but showing an IL-10-dependent inability to induce proinflammatory IL-12.

Previous studies have suggested that intestinal DC may be refractory to stimulation with LPS 3, 12, 13, but we show here that DC from the LP of normal mice constitutively express mRNA for TLR 2, 3, 4, 5 and 9. In addition, similar or greater numbers of mucosal DC expressed surface TLR 2 and 4 and intracellular TLR 3 or 9 proteins compared with DC in spleen or MLN. The expression and function of TLR in LP DC varied with the phenotypic subset, with more CD11c^lo^ class II MHC^lo^ DC expressing all TLR than those with the CD11c^hi^ class II MHC^hi^ phenotype. In parallel, although the CD11c^hi^ class II MHC^hi^ DC expressed higher levels of costimulatory molecules and CCR7 under resting conditions, these molecules were only up-regulated by TLR ligation in the CD11c^lo^ class II MHC^lo^ subset. The nature of this TLR expressing, CD11c^lo^ class II MHC^lo^ subset remains to be determined, with one possibility being that they represent recently recruited immature DC, whereas the CD11c^hi^ class II MHC^hi^ cells have the phenotype of classical mature DC. It should be noted that TLR expression was also dependent on whether LP DC expressed CD103, with much higher numbers of CD103^–^ DC being positive for all TLR than their CD103^+^ counterparts. This was consistent with the fact that most CD103^+^ DC were also CD11c^hi^ class II MHC^hi^ and it also supports other recent findings that CD103^–^ DC in MLN express higher levels of mRNA for TLR 2 and 4 than the CD103^+^ subset 22. As recent work suggests that CD103^+^ and CD103^–^ DC in the gut may be independent subsets with distinct functions (22 and our unpublished observations), it is also feasible that the CD11c^lo^ and CD11c^hi^ subsets of LP DC may not simply represent different stages of maturation of the same cell lineage. Notably, a very recent report has proposed that a significant number of the CD11c^lo^ cells found in mouse small intestinal mucosa, which fail to produce pro-inflammatory mediators in response to TLR ligation may be macrophage-like, based on the expression of F4/80 20. However, our CD11c^lo^ TLR^+^ LP cells appear to be different to these cells, as they are mostly CD11b^neg^F4/80^neg^ and class II MHC^lo^, in contrast to the CD11b^+^F4/80^+^ and class II MHC^hi^ phenotype reported by Denning *et al.* In contrast, the smaller population of F4/80^+^ cells in our preparations were mainly CD11c^hi^ and expressed low levels of TLR. The reasons for these discrepancies are unclear, but could reflect differences in isolation procedures or in the microbial flora present in the different animal facilities. We are now exploring the lineage-function relationships of the different phenotypic subsets of TLR expressing LP DC in more detail.

The proportions of TLR expressing LP DC also varied depending on the anatomical location, with the numbers increasing markedly going down the intestine and being maximal in the colon and distal small intestine. As these are the sites with the highest bacterial load, these findings indicate that TLR expression by intestinal DC may be regulated by the local flora. An identical pattern of TLR 2, 4 and CD14 mRNA expression has been reported at different sites of the mouse intestine, although this study mainly analysed mucosal homogenates and could only locate TLR 4 on epithelial cells and unidentified mucosal mononuclear cells 30. Our work suggests these latter cells may have been DC. Ortega-Cava *et al.* 30 finding that MyD88 was expressed throughout the small and large intestine and our evidence that individual TLR are present on DC from all parts of the intestine contrast with a recent report that LP DC from mouse small intestine do not express TLR 4 mRNA 24. Human intestinal DC also express TLR 4 13 and as our purified CD11c^+^ DC responded to stimulation with the TLR 4 ligand LPS, these results indicate that functional TLR 4 is indeed expressed by mucosal DC. While we were able to confirm previous work that LP DC express high levels of TLR 5 mRNA 24, we were unable to obtain a suitable antibody to confirm expression of the TLR 5 protein and this requires further investigation.

Our study is first to have identified intracellular TLR 3 and 9 in mucosal DC, although TLR 9 has been found previously in intestinal epithelial cells and in uncharacterised LP cells 31, 32. In our hands, TLR 9 was expressed predominantly by the subset of LP DC with the PDCA-1^+^B220^+^ phenotype of plasmacytoid DC, as has been reported in other tissues 11, Others have recently shown a small population of plasmacytoid DC in mouse LP 33 and the potential importance of these cells is demonstrated by the fact that TLR 9 is needed for protective immunity during small intestinal toxoplasmosis in mice 32 and to protect mice from DSS-induced colitis 34. Unlike TLR 2- and TLR 4-mediated responses, we found that TLR 9 (and TLR 3) functioned in an acidic intracellular compartment, as has been described before 11. However, as with the other TLR, stimulation of TLR 9 on LP DC with CpG motifs produced only partial activation, with in particular, no production of the IL-12 which characterises TLR 9 responses by other DC 35. It will be of interest to examine whether the refractoriness to TLR 9 stimulation also affects other characteristic downstream effects, such as type 1 IFN production. In our previous work, we found that LP DC expressed mRNA for type 1 IFN constitutively, but this was not increased further by stimulation with LPS, perhaps consistent with TLR unresponsiveness 3. However, in these experiments, we did not examine TLR 9 stimulation of LP DC and it will be important to explore the basis of this phenomenon more precisely.

The global refractoriness in TLR responsiveness we have identified is clearly distinct from the phenomenon of classical endotoxin tolerance in which chronic exposure of macrophages to BLP, LPS or CpG leads to selective down-regulation of the cognate TLR 36–38. However, cross tolerance has also been described, in which exposure to one ligand inhibits downstream signalling from multiple receptors without affecting their expression 36,40. A generalised phenomenon of this kind might be expected in the intestine, where LP DC are likely to be exposed continuously to multiple TLR ligands. Cross tolerance has been associated with the fact that many of the TLR link with MyD88, TRAF-6 and IRAK-1 adapter proteins and tolerance to LPS causes TLR 4 to fail to recruit MyD88 41–43. However, this mechanism appears to spare responses to TLR 3 and in some cases to TLR 4, when TRIF and TBK-1 are used as adapter proteins to induce downstream signalling 11, 44, 45. Our finding that LP DC were also refractory to stimulation by the TLR 3 ligand poly (I:C) suggests a different mechanism of hyporesponsiveness and we propose that IL-10 may explain at least part of this phenomenon. As we showed previously 3, we found here that LP DC produced significant levels of IL-10 constitutively and now demonstrate that blocking IL-10 signalling allowed these cells to produce IL-12 when stimulated *via* TLR. Inhibition of pro-inflammatory cytokine production by IL-10 is well known and IL-10 can replicate many of the global effects of endotoxin tolerance in myeloid cells, a phenomenon which can be partly prevented by neutralising IL-10 46. IL-10 may act by interfering with the remodelling of the IL-12 p40 gene locus and/or by inactivating a subset of p40 transcriptional activators 28. Interestingly, IL-10-induced TLR hyporesponsiveness is associated with maintained receptor expression, further supporting the idea that IL-10 may be responsible for the functional defects we observed in LP DC.

Together, our results support the idea that autocrine production of IL-10 by mucosal DC may underlie their inability to respond fully to TLR and it is likely that this default IL-10 response reflects conditioning effects of the local microenvironment 47. This could involve the large amounts of TGFβ found in the gut and a similar population of IL-10-producing DC has been described after conditioning with TGFβ-producing splenic stromal cells 48. This phenomenon is related to TGFβ-mediated activation of ERK1/2 and failure to activate p38 in response to TLR. Alternative conditioning factors could include TSLP released by intestinal epithelial cells 49, 50, or PGE2 released by TLR-activated mucosal stromal cells 51, 52. Additionally, initial stimulation of TLR on immature DC arriving in the mucosa could prime for selective production of IL-10 and inhibition of IL-12 on subsequent encounters with PAMP, as has been shown with TLR 2 or 4 *in vitro* 53. This process is also dependent on selective activation of ERK1/2. That this may occur in the normal gut is supported by the finding that individual commensal bacteria can also prime IL-10 production by immature DC *in vitro* 54, 55, thus setting up a feedback loop with IL-10 at its core.

In summary, our results are consistent with the hypothesis that LP DC are conditioned by intestinal bacteria and other local factors to develop IL-10-dependent unresponsiveness to inflammatory stimuli such as TLR ligands. We propose that this allows LP DC to play a central role in maintaining homeostasis to dietary proteins and commensal bacteria. Several populations of intestinal DC have been shown to produce IL-10 and in PP this is associated particularly with the CD8α^–^ subset 56. In the LP, it has been suggested that IL-10 production is a selective property of the CD11c^lo^F4/80^+^ “macrophage-like” subset 20, but we found that neither CD11c^lo^ nor CD11c^hi^ LP DC produced IL-12 in response to TLR stimulation. Therefore, we are currently investigating the role of the individual phenotypic subsets in the production of IL-10. It will be of particular interest to explore the role of the CD103^+^ subset in this phenomenon, as these cells in LP and MLN have recently been shown to have a selective ability to induce the generation of FoxP3^+^ Treg *in vitro* 21, 22. This requires TGFβ and retinoic acid, but the role of IL-10 has not yet been explored. Importantly, we found that despite their partial refractoriness to activation, LP DC expressed significant levels of CCR7 and this was enhanced by TLR stimulation. CCR7-expressing DC also migrated *in vitro* in response to CCL19, indicating that they would have the capacity to emigrate to the draining MLN *in vivo* even under non-inflammatory conditions. This is consistent with the considerable trafficking of mucosal DC into lymph, which occurs in resting animals 50, and with the evidence that constitutive migration of resting tissue DC to LN is a central component of self-tolerance 57. More specifically, recent studies show that the induction of tolerance to orally or intranasally administered antigens requires CCR7-dependent migration of antigen-loaded DC from the gut mucosa or lung parenchyma to the draining LN 6, 58. Our data suggest that IL-10-dependent refractoriness of mucosal DC allows this crucial homeostatic process to be maintained in the intestine, allowing uptake and presentation of harmless antigens to T cells in organised lymphoid tissues, but ensuring tolerance is the result.

## Materials and methods

### Mice

Female BALB/c mice were obtained from Harlan Olac (Bicester, UK). All mice were first used at 8 weeks of age and were maintained under SPF conditions at the Common Research Facility of the University of Glasgow.

### Treatment of mice with flt3 ligand

Mice were injected daily i.p. with 10 μg recombinant human flt3L (kindly provided by Amgen, Seattle, WA) in saline for 7–10 days before harvest of DC.

### Isolation of cells from lymphoid tissues

Single-cell suspensions were prepared from MLN and spleen either by gently mashing through nylon mesh filters (Becton Dickinson, BD; Cowley, UK), resuspended in RPMI 1640 (Life Technologies, Paisley, UK) or by digestion for 20 min at 37°C with 100 U/mL collagenase (type VIII; Sigma, Poole, UK) and 30 μg/mL DNase I (Roche Diagnostic, Lewes, UK) in calcium- and magnesium-free HBSS (CMF; Life Technologies) containing 10% FBS (Life Technologies). The cells were passed through Nitex mesh, counted by phase contrast microscopy and kept in RPMI with 10% FBS.

### Isolation of LP cells

Small intestines and colons were washed in HBSS and the PP excised. To obtain LP cells, the guts were opened longitudinally, cut into small pieces and washed thoroughly in HBSS before being incubated in HBSS containing 2 mM EDTA (Sigma) for 15 min in a shaker at 37°C. The epithelial layer was then removed by shaking the pieces of intestine twice thoroughly in HBSS and the incubation process repeated for four cycles. The remaining fragments of intestinal tissue were then incubated with 100 U/mL Collagenase and 30 μg/mL DNase I in RPMI/10% FBS at 37°C for 45 min. The fragments were then disrupted by shaking and the supernatants collected. After repeating the process three times, supernatants were passed through Nitex mesh, washed and stored in RPMI/10% FBS.

### Purification of DC

CD11c^+^ DC were purified from the intestine and spleen by MACS according to the manufacturer's instructions (Miltenyi Biotec). Briefly, cells were incubated with MicroBeads conjugated anti-CD11c (N418, Miltenyi Biotec) antibody and suspended in cold MACS buffer (PBS + 0.5% BSA + 2 mM EDTA) for 15 min at 4°C and passed over MD MACS column (Miltenyi Biotec). Positively selected cells were routinely found to contain 82–91% CD11c^+^ cells by flow cytometry.

For sorting of DC into CD11c^hi^ and CD11c^lo^ subsets, LP cells were first incubated with FITC-conjugated anti-CD11c mAb (HL3, Hamster IgG; BD Biosciences) and then sorted on a FACSVantage (Becton Dickinson) into CD11c^hi^ and CD11c^lo^ fractions. The purity of the sorted DC subsets routinely exceeded 90% and 80%, respectively.

### Stimulation of DC with TLR ligands

To examine the response of DC to TLR stimuli, DC were resuspended in RPMI/10% FBS and cultured at 1 × 10^6^ cells/mL in 24-well ultra-low attachment polystyrene plates (UltraLow Cluster, Corning, Corning, USA) with 2 μg/mL BLP (*Pam3CSK*; EMC Microcollection, Tubingen, Germany), 5 μg/mL poly (I:C) (Invitrogen), 2 μg/mL Flagellin (*Salmonella T*.; Invitrogen), 5 μg/mL LPS (*E. coli,* Sigma) or 5 μM CpG (ODN 1826, Invitrogen), for 4, 6, 8, 12 or 24 h at 37°C. In some experiments, DC were pretreated with chloroquine (2.5 μg/mL for 15 min, Sigma) or with anti-IL-10R (2 ng/mL for 60 min, 1B1.3a, BD PharMingen) or the rat IgG control antibody before stimulation with TLR ligands.

### Flow cytometry

Aliquots of spleen and intestinal DC were stained with PE, FITC or biotinylated anti-CD11c (HL3, BD PharMingen) in combination with FITC- or PE-antibodies against CD80 (16–10A1, BD PharMingen), CD86 (GL1, BD PharMingen), MHC class II (I-A^b^ 25-9-17, BD PharMingen), B220 (RA3–6B2, BD PharMingen), TLR-2 (6C2, E Bioscience), TLR-3 (T3.7C3, E Bioscience), TLR-4 (MTS510, E Bioscience), TLR-9 (M9.D6, E Bioscience), CCR7 (4B12, E Bioscience), IL-12(p40/p70) (C15.6, BD PharMingen), F4/80 (BM8; Caltag Laboratories), CD11b (M1/70, BD Bioscience). Appropriate isotype-matched controls from BD PharMingen were included in all experiments. Biotinylated antibodies were detected using streptavidin conjugated to allophyocyanin (APC; BD PharMingen), and all staining procedures were carried out using 1 × 10^5^ to 5 × 10^5^ cells in 200 μL FACS buffer (PBS + 2% FBS and 0.05% sodium azide) for 30 min at 4°C in the dark. For intracellular staining cells were fixed with 1% formaldehyde for 10 min and subsequently permeabilized with 0.5% saponin in 1% BSA FACS buffer, prior to staining with anti-TLR 9, TLR 3 or IL-12 antibody. To exclude dead cells, 5 ng propidium iodide (PI; Sigma) was added just before analysis and cells were analyzed using a FACSCalibur cytometer and CellQuestPro software.

### Measurement of cytokine production *in vitro*

After 24 h of culture in medium or with TLR ligands, DC supernatants were harvested, centrifuged and stored at –70°C. Cytokine production was quantified using sandwich ELlSA techniques as previously described 59. Cytokine concentrations in test supernatants were determined with reference to a standard curve constructed using serial dilutions of the standard cytokines 59.

### Assessment of chemotaxis *in vitro*

Total LP cells were resuspended at a final concentration of 2.5 × 10^6^/mL cells and cultured in 24-well tissue culture plates (Costar) either in medium alone, or with the different TLR ligands for 6 h. Of the cells suspension, 200 μL was then added to the upper chambers of a Transwell chemotaxis plate (5-μm pores; Costar) and 400 μL of serum-free RPMI1640 containing 10 ng/mL CCL19/MIP3β (PeproTeck) was placed in the lower chamber. The plate was then incubated at 37°C for 2 h and the cells that had migrated into the bottom chamber were stained with a FITC-anti-CD11c and counted using a FACSCalibur cytometer and CellQuestPro software.

### Analysis of mRNA expression by RT-PCR

Total RNA was extracted from purified small intestinal LP DC and spleen using the RNeasy Mini Kit extraction (Qiagen) according to the manufacturer's instructions. The mRNA levels were quantitated by absorbance at 260 nm and cDNA was synthesized from 5 μg of total RNA using 200 U of superscript II reverse transcriptase, 2.5 μmol/L random hexamers, 1 μmol/L deoxynucleoside and 40 U/mL of ribonuclease inhibitor (all from Invitrogen) in a total volume of 25 μL. The reaction was performed at 42°C for 50 min.

RT-PCR reactions were performed in a 1.1x Pre-aliquoted ReddyMix PCR Master Mix 50 µL Reaction (ABgene) used as the manufacturer's instructions with 25 pmol/L 5′ and 3′ primers and 2 μL cDNA. Reactions were incubated in FTGENE5D thermocycler (Teche) for 35 cycles (denaturation 1 min at 94°C, annealing for 1 min at 60°C, and extension for 1 s at 72°C) using the following PCR primers: TLR 2 (5′-CGTTGTTCCCTGTGTTGCT-3′, 5′-AAAGTGGTTGTCGCCTGCT-3′); TLR 3 (5′-TTGCGTTGCGAAGTGAAG-3′, 5′-TAAAAAGAGCGAGGGGACAG-3′); TLR 4 (5′-TTCACCTCTGCCTTCACTACA-3′, 5′-GGGACTTCTCAACCTTCTCAA-3′); TLR 5 (5′-CAGGATGTTGGCTGGTTTCT-3′, 5′-CGGATAAAGCGTGGAGAGTT-3′); TLR 9 (5′-GAAAGCATCAACCACACCAA-3′, 5′-ACAAGTCCACAAAGCGAAGG-3′); GAPDH (5′-AACTCCCACTCTTCCACCTT-3′, 5′-GCCCCTCCTGTTATTATGG-3′). Of RT-PCR products, 10 μL was combined with 1 μL of loading buffer and electrophoresed on a 2% agarose gel (in 0.5x TBE buffer: 0.275% Boric acid, 2 mM EDTA, 0.54% Tris base). Gels were analysed using the Gel Logic 200 imaging system.

### Statistical analysis

Where appropriate, results were expressed as means ± 1 SD and differences between groups were compared using Student's *t*-test.

## Abbreviations

BLP: bacterial lipoprotein
LP: lamina propria
pDC: plasmacytoid dendritic cell
PP: Peyer's patches
